# Multiclinic Observations on the Simplified Diet in PKU

**DOI:** 10.1155/2017/4083293

**Published:** 2017-09-13

**Authors:** Laurie Bernstein, Casey Burns, Melissa Sailer-Hammons, Angela Kurtz, Frances Rohr

**Affiliations:** ^1^Inherited Metabolic Diseases Clinic, Children's Hospital Colorado, Aurora, CO, USA; ^2^Metabolic Nutrition Program, Division of Medical Genetics, Icahn School of Medicine at Mount Sinai, New York, NY, USA; ^3^Department of Nutrition, Boston Children's Hospital, Boston, MA, USA

## Abstract

Phenylketonuria is an inborn error of metabolism that historically has been treated with a strict phenylalanine-restricted diet where all foods are weighed and measured. This is cumbersome and difficult for patients and caregivers, especially patients with high phenylalanine blood concentrations who often have neurocognitive deficits. The Simplified Diet is an alternative approach that allows for increased flexibility, promotes healthy food choices, and is easier to manage than a traditional diet for PKU. This paper describes the implementation of the Simplified Diet and outlines education, counseling strategies, and challenges encountered by three metabolic clinics in the United States.

## 1. Introduction

Phenylketonuria (PKU) is an inborn error of metabolism that causes mutations in the phenylalanine hydroxylase (PAH) gene [1]. PAH catalyzes the conversion of phenylalanine into tyrosine with the help of the cofactor tetrahydrobiopterin (BH4) [2]. A deficiency in PAH causes the accumulation of phenylalanine in the blood and other tissues, including the brain, and results in intellectual disability if left untreated. The primary treatment for PKU is a lifelong diet that consists of a prescribed amount of phenylalanine and is aimed at keeping blood phenylalanine in the treatment range of 120–360 *μ*mol/L [3]. Patients with a severe or classical form of PKU are prescribed approximately 250–300 mg of phenylalanine per day [4], which is equivalent to about 5-6 grams of protein from food. Allowed foods include fruits, vegetables, fats and oils, sugars, and modified low protein foods (e.g., low protein bread, pasta). High protein foods such as meat, fish, eggs, dairy products, nuts, and legumes must be avoided. The majority of an individual's protein requirements are met by medical foods designed for PKU that contain amino acids other than phenylalanine, as well as vitamins and minerals. Adjunct treatment with sapropterin dihydrochloride (synthetic BH4) causes a reduction in blood phenylalanine in about 50% of individuals with PKU [5] and allows for 50% higher intake of protein-containing foods in the diet, on average [6]; however, the vast majority of these individuals still require medical food and intact protein restriction to keep blood phenylalanine in the treatment range.

Many patients have difficulty adhering to the diet recommendations [7] due the extent of the dietary protein restriction, lack of access or acceptance of modified low protein foods, poor palatability of medical foods, and cost [8]. Following the diet carefully requires the individual with PKU or their caretakers to strictly monitor phenylalanine intake from food. Traditionally, the diet has been managed by weighing and measuring all foods eaten, looking up the phenylalanine content of each food, and keeping an exact record of dietary intake. For individuals who have high blood phenylalanine concentrations, following the diet is even more difficult due to the neurocognitive problems associated with high blood phenylalanine concentrations, including executive function deficits, anxiety, and slow processing speed [9], which can interfere with choosing and measuring foods appropriately [8]. Following the diet can be even more difficult for individuals who have blood phenylalanine concentrations above treatment range, due to the association between high blood phenylalanine and neurocognitive problems. Executive function deficits, anxiety, and slow processing speed [9] can interfere with the skills needed to adhere to the diet, such as choosing and measuring foods appropriately [8]. In addition, counting and tracking phenylalanine intake add to the burden of managing the diet for PKU [10].

The Simplified Diet method of managing dietary phenylalanine intake in patients with PKU has been studied in Europe and Australia. In a 2003 study, 15 subjects with PKU from the United Kingdom consumed different amounts of “free” foods during 3 study phases. Free foods in Phase 1 included fruits and vegetables containing less than 50 mg phenylalanine/100 g; in Phase 2: fruits and vegetables containing less than 75 mg phenylalanine/100 g; and in Phase 3, fruits and vegetables containing less than 100 mg phenylalanine/100 g of food. Blood phenylalanine and diet intake were monitored for 15 weeks and there was no effect on plasma phenylalanine concentrations during all three phases [11]. Another study, in which subjects consumed foods containing 100 mg of phenylalanine/100 g without counting these foods, showed no negative effects on plasma phenylalanine concentrations [12]. However, during this study, fruits and vegetable consumption did not increase. Rohde et al. conducted three studies (lasting 4 weeks, 1 year, and 3 years) where “free” foods were defined as those containing less than 75 mg of phenylalanine/100 grams of food. While total phenylalanine intake increased slightly, there was no significant difference in blood phenylalanine concentrations when counting with this method [13, 14]. In Australia, good metabolic control was associated with a phenylalanine-counting method where foods with less than 50 mg phenylalanine/100 g were considered free and other foods were counted using 0.5 g increments in protein [15].

The Simplified Diet is defined here as an approach to managing the PKU diet that allows individuals with PKU to consume foods that contain lower amounts of phenylalanine without measuring or counting them, recognizing that no diet for PKU is simple. It is designed to provide increased flexibility, promote healthy food choices, and be easier diet management than the traditional method of counting all phenylalanine consumed. While the counting method is different in each clinic, the goal of the Simplified Diet is the same: to maintain blood phenylalanine in the recommended treatment range of 120–360 umol/L. In many clinics in the United States, the Simplified Diet is a new approach to managing PKU, but in others, it has been used for years but was called the Low Protein Diet. This paper aims to describe research on the effectiveness of the Simplified Diet and outlines education, counseling strategies, and challenges encountered in implementing the diet by registered dietitians in three US clinics.

## 2. Materials and Methods

This manuscript includes the experiences of five registered dietitians at three metabolic clinics in the US: one that began using the Simplified Diet in 2015, one that has been using this approach since 1965, and one that has used this method with adults returning to diet since 1983, but only recently for all patients with PKU.

## 3. Results and Discussion

**Figure 1:**
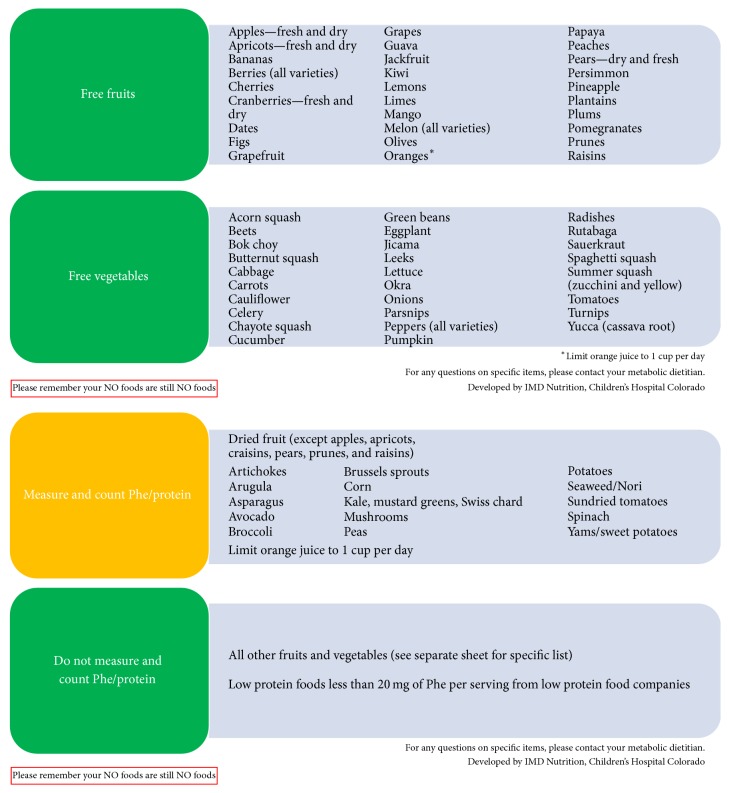
Simplified PKU diet; Inherited Metabolic Diseases Clinic, Children's Hospital Colorado.

Experiences regarding the effectiveness of the Simplified Diet, education, counseling strategies, and challenges encountered in implementing the diet were collected and are described. While the foods counted or allowed as free vary slightly from clinic to clinic (Figure 1 and Supplementary Materials available online at https://doi.org/10.1155/2017/4083293), the concept of only counting certain foods while allowing others to be consumed freely is the same. The mainstay of diet treatment is medical food that contains little or no phenylalanine and provides the majority of protein to patients with PKU.

### 3.1. Transitioning to the Simplified Diet

In one clinic, all patients who had counted in the traditional fashion were transitioned to the Simplified Diet. Families and patients were provided with a letter detailing the history of the Simplified Diet, how the trial period would work, and what diet changes would be made. The concept was first presented in a group setting with a pilot group of 15 patients and allowed for parents and patients to openly discuss concerns and ask questions for clarification. The main concern expressed by families was how fluctuating intake would affect blood phenylalanine concentrations. All patients/families present chose to implement the Simplified Diet following the clinic visit.

Prior to starting the new counting method, blood phenylalanine was measured. Each patient's phenylalanine prescription was reduced by 30%. For example, if a patient was previously prescribed 300 mg of phenylalanine/day, when transitioning to the Simplified Diet, they were counseled to count 210 mg of phenylalanine, but no longer to count or measure certain free fruits or vegetables that contain less than 75 mg of phenylalanine per 100 grams of food. Low protein modified foods containing less than 20 mg of phenylalanine per serving were also considered free and did not need to be counted.  Table 1 is an example of a diet containing 300 mg of Phe and compares what is counted using a traditional method and the Simplified Diet method.

Families were provided with detailed handouts stating which fruits, vegetables, and low protein foods could be eaten freely (Figure 1). Weekly blood phenylalanine was measured and results were overall stable. Families reported increased satisfaction with the diet and more independence in food choices for the patient with PKU. Older children, teens, and adults who previously had to track intake of all foods often chose fewer fruits and vegetables because they wanted to use their phenylalanine “allowance” for foods such as potatoes and snack foods.

No diet changes were made during the 4-week trial period, even if blood phenylalanine concentrations fluctuated slightly. This allowed for the “newness” of the diet to subside. When first allowed the option to eat certain foods freely, some patients ate more than the usual amount of some foods but after several weeks' intake returned to typical volume. Following implementation of the diet with the pilot group, the letters and handouts specifying the free fruits, vegetables, and low protein foods were sent to all patients in the clinic. All families that are seen regularly in the clinic now follow the Simplified Diet, a transition that took approximately two years.

### 3.2. Implementing the Simplified Diet: Infants

When an infant is 4–6 months of age, parents are instructed to offer only free foods, at first, so that the child can become accustomed to eating solids before any foods must be counted. As the child accepts free fruits and vegetables, counted foods are introduced, typically dry infant cereal or a counted vegetable, such as spinach or sweet potato. In one clinic, parents are counseled to start counting protein from dry cereal after the infant can consume two tablespoons of dry cereal per day. At that point, a one-gram protein diet is initiated, followed by increments in protein intake of 0.5 to 1 gram of protein, with a corresponding decrease of protein from standard infant formula. For clinics where phenylalanine is counted, typically 15 mg increments of phenylalanine from food are allowed. Concomitant with the introduction of counted foods, the dietitian must decrease the amount of phenylalanine from standard infant formulas. To account for the free foods that will be eaten, the dietitian must reduce the phenylalanine content of medical food/infant formula recipe by more than 15 mg of phenylalanine from the infant's formula. For example, 20 mg of phenylalanine would be reduced in the infant formula but the parents would count only 15 mg of solid food intake (30% of total phenylalanine intake accounted for by free foods). For breastfed infants, no adjustment is routinely made to the medical food. Intake of breast milk typically declines when food portions increase and blood phenylalanine is closely monitored. If an increase in blood phenylalanine is seen, the volume of medical food recommended is increased. Breastfeeding is encouraged in a way that does not interfere with mealtime or medical food intake.

With the Simplified Diet, families of infants and toddlers report being able to introduce new foods without worrying about the phenylalanine that may not be consumed due to finicky eating, food spills, or incomplete intake.

### 3.3. Adults Returning to Treatment

In one clinic, the simpler approach to managing the diet was first used with adults and maternal PKU patients returning to clinic who were treated as children and taken off treatment at about 5 or 6 years of age. The simplified approach to counting phenylalanine was born out of necessity as it was overly burdensome and unrealistic for patients who had been on an unrestricted diet for decades to start to measure and record everything they ate. These adults had never learned to manage the diet themselves as children, because they were taken off diet at a young age. Moreover, when returning to clinic, the adult patients often had the neurocognitive deficits associated with having high blood phenylalanine concentrations for many years that made learning and following the diet very difficult. When this approach was first used, the target blood phenylalanine was 120–600 *μ*mol/L. However, the approach was also taught to women returning to the diet for pregnancy where target blood phenylalanine was 120–360 *μ*mol/L. For women with PKU who came to attention already pregnant, there was an inordinate amount of information to learn in a short period of time in order to protect the fetus: choosing and preparing medical food, accessing medical food and low protein food, cooking low protein foods, monitoring blood phenylalanine, and tracking food intake. Simplifying the counting of phenylalanine was essential, practical, and effective in maintaining good metabolic control. Adults using the Simplified Diet have expressed that they enjoy freedom to choose foods when eating outside of the home at school, while socializing with friends, or at work.

### 3.4. Challenges

Challenges have been minimal. While the simplified method has been well accepted, implementing the Simplified Diet has been more of a challenge with parents who have followed the diet carefully for many years and, at first, have been resistant to change. They sometimes have difficulty “letting go” of counting and tracking all food intake. However, with time, most parents see the benefit of having their child expand daily consumption of fruits and vegetables while maintaining blood phenylalanine in the treatment range. The ability to choose freely from the foods that are not counted allows a greater sense of independence and more flexibility, especially as children age.

One challenge has been for patients who have a very low tolerance to phenylalanine, less than 250 mg/day. For these individuals, frequent intake of the free foods that are toward the high end of phenylalanine content (those that are close to the 75 mg phenylalanine/100 g cut-off) may cause elevations in blood phenylalanine, and the ratio of free foods to counted foods must be modified for these patients. Usually the phenylalanine prescription is divided into 30% of phenylalanine from free foods and 70% from counted foods, but for the patient with very low phenylalanine tolerance, 40% of the prescription must be “set aside” for free foods. If blood phenylalanine concentrations start to increase, diet records are kept and analyzed to determine if free food intake exceeds 30%. Conversely, patients with a higher phenylalanine tolerance, especially patients with mild or moderate PKU or those who respond to sapropterin dihydrochloride, can often choose several foods with relatively high phenylalanine content from the “free” foods on a particular day and yet have little variation in blood phenylalanine. For these individuals, 20–25% of the prescription is set aside for free foods.

For individuals that have been following this method for a long time, measuring of food portions may become too relaxed, the intake of higher protein vegetables may become excessive, and/or the quantity of low protein foods may increase, causing excessive protein and phenylalanine intake and elevated concentrations of blood phenylalanine. Regardless of the method used to count intake, proper management of the PKU diet requires close monitoring of blood phenylalanine and adjusting the diet as necessary to keep blood phenylalanine in the recommended treatment range.

## 4. Conclusion

This paper offers perspectives from three US clinics that have implemented the Simplified Diet. While the approaches to implementing the diet vary slightly, all have observed that the Simplified Diet is easier to follow, encourages healthy food choices, and can improve the quality of life for patients with PKU as compared to the traditional counting method. Since adherence to the diet for PKU is poor, especially in older teens and adults, strategies to simplify the diet should be considered. Research on the long-term nutrient intake and metabolic control of patients on the Simplified Diet is needed.

## Supplementary Material

Image 1: The Simplified Diet for PKU Boston Children's Hospital, Boston MA.
Image 2: The Simplified Diet for PKU Icahn School of Medicine at Mount Sinai, New York, NY.

## Figures and Tables

**Table 1:** Example of counting phenylalanine using the traditional counting method and the Simplified Diet^1^.

Food eaten	Traditional counting method	Simplified diet
Apple Jacks® Cereal^2^, 1/2 cup	40 mg	40
Low protein bread, 2 slices	30 mg	Free
Low protein cheese, 2 slices	30 mg	30 mg
Pringles®^2^, 3 each	15 mg	15 mg
Apple, 1	10 mg	Free
French fries	120 mg	120 mg
Low protein pasta, 1 cup	20 mg	Free
Green beans, 1/2 cup	35 mg	Free
Medical food, 24 ounces	0	0

Total	300	205

^1^In this example, the patient's phenylalanine tolerance is 300 mg/day; the patient counts 210 mg from food and is allowed unlimited “free foods” (defined as those containing <75 mg phenylalanine/100 g food as well as low protein foods with less than 20 mg Phe per serving). ^2^Kellogg Company, Battle Creek, MI, USA.
