# Supplementary material for: Multiclinic Observations on the Simplified Diet in PKU
Source: J Nutr Metab. 2017 Sep 13;2017:4083293. doi: 10.1155/2017/4083293 (PMC5615976; doi:10.1155/2017/4083293)
Supplement: Supplementary file 1 — Image 1: The Simplified Diet for PKU Boston Children's Hospital, Boston MA. Image 2: The Simplified Diet for PKU Icahn School of Medicine at Mount Sinai, New York, NY. [file 4083293.f1.pdf]

Supplementary Materials

Image 1. Simplified PKU Diet; Department of Nutrition, Boston Children's Hospital

Simplified PKU Diet

Medical Food (Formula)

- Medical food provides protein that is phenylalanine-free or low in phenylalanine.
- Medical food takes the place of high protein foods such as meat and dairy products

Food

- The diet includes foods that are low in protein/phenylalanine.
- Count intake using the guidelines below.
- Recommended intake ___________________

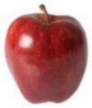
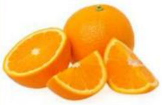

<u>Low Protein Foods - No Limit</u>

Fruits

- All fruits that are fresh, frozen, or canned.
  Examples: apple, orange, pear, peach, melon, banana, pineapple, nectarine, berries, plum, kiwi, mango, grapefruit, clementine, fruit cocktail, applesauce, grapes
- Dried cranberries, raisins, prunes, and apples.

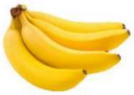
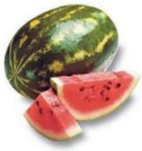
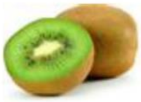
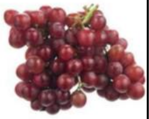

Vegetables

- Most are low in protein – no limit
  Examples: asparagus, brussels sprouts, carrot, celery, cucumber, lettuce, onion, beet, cabbage, green pepper, red pepper, zucchini, cauliflower, squash, mushrooms, green beans, wax beans, artichokes, eggplant, tomato, plantain, raw spinach, raw arugula, and raw kale

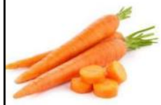
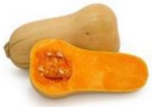
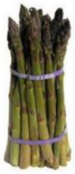
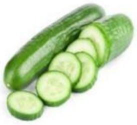

Specialty Low Protein Foods

- Most are low in protein*
  Examples: low protein pasta, rice, bread, bagel, cereal, broth, cookies, peanut butter substitute

*Discuss with dietitian about where to order, which to count

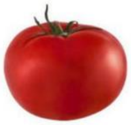
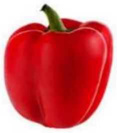
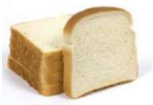
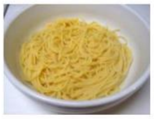

Other Foods

- Contain sugar and fat, but no protein, add extra calories
- Examples:
  - Soft drinks such as lemonade, soda, iced tea (avoid aspartame)
  - Fats – butter, margarine, vegetable oil, mayonnaise, salad dressings (if label indicates zero protein)
  - Other – nondairy creamer, coconut milk yogurt and ice cream; jelly, jam, sugar, maple syrup, popsicle, Italian ice, sorbet, marshmallow, hard candy

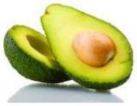
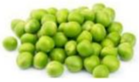
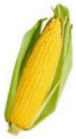
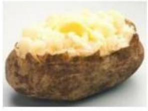
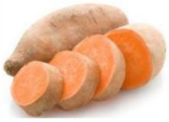
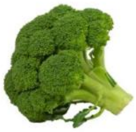

Medium Protein Foods – Need to Count

These vegetables/fruits are higher in protein and must be counted

Artichokes	Potatoes
Arugula (cooked)	Portabella Mushroom
Avocado	Seaweed
Broccoli	Sundried tomatoes
Collards (cooked)	Sweet Potatoes
Corn	Swiss Chard
Kale (cooked)	Yam
Peas	

Dried fruits (e.g. dried apricots, bananas, dried cherries)

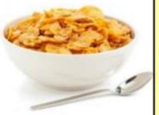
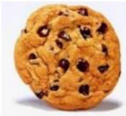
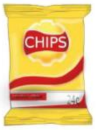

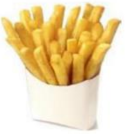

Bread and Cereal Products

- Read food labels- look for the lowest in protein
  Examples:
  - Breakfast foods: toast, bagel, English muffin, waffle, pancake, cereal, small muffin, donut
  - Lunch and dinner foods: bread, pita, roll, tortilla, rice, pasta
  - Snack foods: potato chips, corn chips, rice cakes, Goldfish, crackers, popcorn, cookies

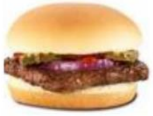
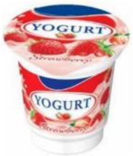

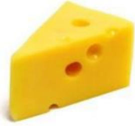
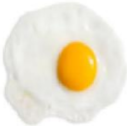

High Protein Foods (Avoid)

- These foods are high in protein
  - Meat (All types- beef, lamb, pork, chicken, turkey, fish, etc.)
  - Eggs
  - Dairy products (cheese, yogurt, ice cream)
  - Nuts and seeds (including nut/seed butters)
  - Soy protein (tofu tempeh, many vegetarian burgers)
  - Legumes (kidney beans, garbanzos, lentils, split peas, hummus)

Boston Children's Hospital Metabolism Program
Fran Rohr, MS RD
Ann Wessel, MS, RD
Leslie Martell, MS, RD

Rev. 10/11/2016

Image 2. Explanation of Low Protein Diet; Metabolic Nutrition Program, Division of Medical Genetics, Icahn School of Medicine at Mount Sinai

Icahn School of Medicine at Mount Sinai

Department of Genetics and Genomic Sciences
Icahn Institute for Genomics and Multiscale Biology

METABOLIC NUTRITION PROGRAM

Explanation of Low Protein Diet

The low protein diet is divided into 4 categories:

1. Foods not allowed (completely eliminated from diet)
2. Foods allowed in limited quantities, called "limited items"
3. Foods allowed in unlimited quantities (as much as desired)
4. Special formula

1. Foods not allowed

- All Meat and meat products (includes gelatin and bouillon)
- Fish, fish products, and all types of seafood.
- Milk in any form, dairy products and all derivatives (such as eggs, cream, cheese, ice cream, sherbet, yogurt, etc.)
- Legumes such as soybeans, lentils, split peas, lima beans, green peas, any peas or beans (except green beans or wax beans), or any isolated protein derived from these sources such as vegetable protein, soy protein, soy flour, soy sauce, soybean curd (tofu), etc.
- Nuts and nut butters (including coconut and chestnuts)
- Seeds (all including sesame seed paste (Tahini).
- Any commercial product or condiments containing protein unless approved by the nutritionist.

2. Foods allowed in limited quantities

- Corn (including popcorn and corn chips)
- Potatoes (including French fries, potato chips, and yams)
- Rice and rice products
- Breads and baked goods
- Commercial breakfast cereals containing less than 2 grams of protein per cup
- Oranges, orange juice, bananas and plantains

The amount of all limited items and foods containing protein must be strictly controlled. Patients may be allowed 1 to 3 limited items or 0-10 grams of protein daily depending on doctor's or nutritionists orders. If blood levels cannot be maintained within a satisfactory range the number of limited items is reduced. Portions of all limited items and any food containing protein must be measured. For the correct potion sizes see Portion Sizes for Limited Items

3. Foods allowed in unlimited quantities

- All vegetables except the ones listed as limited or marked with an asterisk. See **Allowed Vegetables**
- All fruits except the ones listed as limited. See **Allowed Fruits**
- Special low protein products such as wheat starch bread, wheat starch, low protein baking mix, and all products made from these items. Sold as low protein foods from specialty companies.
- All condiments and foods which are free of any food or derivatives of foods which are un-allowed or limited. **See Allowed Condiments** and **Unlimited Foods**
- All beverages which are free of any food or derivatives of foods which are un-allowed or limited. **See Allowed Beverages**

4. Special Formula or Amino Acid Supplement

The special products are prescribed by the metabolic nutritionist to supply adequate protein, calories, vitamins, and minerals for normal growth and development.

ALLOWED FRUITS

acerola
apples
apricots
bananas (limited)
blackberries
cherries
cranberries
currants
dates
figs
grapes (all varieties)
grapefruit
guava
kiwi fruit
kumquat
lemons
limes
lychees
loquat
mangos
melons (all varieties)
nectarines
oranges (limited)
passion fruit
paw paw
papayas
peaches
pears
persimmons
pineapple
plantains (limited)
plums (all varieties)
pomegranate
prunes
quinces "emu apple"
raisins
raspberries
rhubarb
strawberries
sapodillo
tangeloes
tangerines
watermelon

NOTE: Limited fruits must be counted as limited items when large portions are eaten (see Portion Sizes for Limited Items), These fruits do not have to be counted as limited items, however, when used in small quantities such as in cooking (ex: banana bread), in fruit salad, etc.

ALLOWED VEGETABLES

Vegetables marked with an * when eaten in large quantities, add a significant amount of protein to the day's intake. Do not eat more than one vegetable marked with an asterisk per meal, and ½ cup max. They may be used freely in small quantities such as vegetable stews and mixed dishes. The number in parentheses is for protein content in **½ cup, cooked** except for parsley.

acorn squash
anise
*artichoke (2.43 g)
asparagus
*bamboo shoots (0.92 g)
basella (Indian spinach)
beets
beet greens
*broccoli (1.86 g)
broccoli rabe
*brussel sprouts (1.99 g)
burdock
butternut squash
cabbage
carrots
*cassava = yuca, manioc (1.5g)
*cauliflower (1.14 g)
celery
celeriac (celery roots)
chard
chayote squash
chicory
Chinese cabbage (Bok choy)
chives
*collard greens (1.2 g)
coriander (chinese parsley)
corn (limited)
cucumber
dandelion greens
dasheen (Japanese taro)
eggplant (aubergine)
endive
escarole
*fennel (finocchio, 0.54g)
garlic
ginger root
green beans (string beans)
hearts of palm
hubbard squash
horseradish root
*kale (1.24 g)
kohlrabi
leek
lettuce (all varieties)
*lotus root (0.95 g)
*mushrooms (1.13gm, cooked)
mustard greens
okra
olives
onions
*parsley (0.89 g)
parsnips
peppers (red and green)
potatoes (limited)
pumpkin
purslane
radishes
rutabaga
sauerkraut
*salsify (oyster plant, 1.84 g)
scallions
shallots
spinach, raw
spinach, cooked (2.6g)
squash (all varieties)
sorrel (dock, sourgrass)
sweet potato, yams (limited)
tampala leaves
tomatoes
turnips
*turnip greens (0.82 g)
watercress
water chestnuts
wax beans
yautia (mélange, limited)
zucchini

Revised by Angela Kurtz, MS, RD, CDN 9/16/2013

ESTIMATED PORTION SIZES FOR LIMITED ITEMS

Containing 2 grams of protein

1 serving potato =	1 small potato (2 inch diameter)
	1/2 large potato (4-3/4 inch X 2-1/2 inch)
	1/2 cup mashed potatoes (no milk)
	20 potato chips (2 inch diameter) or a 1 - ounce bag
	10 thick French fries (1/2 X 1/2 X 2 inch)
	20 very thin French fries (McDonald's type – small order)
	note: medium = 4 grams and large = 6 grams
1 serving corn =	1 <u>small</u> ear of corn on cob (3" long)
	1/2 cup corn
	1 1-ounce package Fritos corn chips
	1-1/2 tablespoon un popped popcorn (110 kernels = 25.5 g)
	1- 1/2 corn tortilla (6 inch diameter)
1 serving rice =	1/2 cup cooked brown or white rice
1 serving cereal =	1 cup allowed ready-to-eat breakfast cereal
	1 cup cooked rice cereal
1 serving orange =	1- 1/2 medium orange *****
	10 ounces orange juice
	1 cup orange segments
1 serving banana =	2 small bananas (7-3/4 inch X 1-1/4 inch)
	3/4 cup mashed banana
	1-1/4 large banana (9-3/4 inch X 1-1/2 inch)

*****NOTE: Florida oranges contain less protein than California oranges, therefore serving sizes are based on Florida oranges.

These are **estimates**, please **read the food labels on packaging**.

ALLOWED CONDIMENTS/SAUCES

Jelly/Jam
Syrup (all types
Catsup (any brand)
Mustard (any brand)
A-1 Steak Sauce
Horseradish
Imitation butter flavoring
Flavoring extracts
Oil-based salad dressing
Progresso pizza sauce
Buitoni marinara spaghetti sauce
Ragu pizza sauce
Tapioca
Arrowroot
Baking powder
Baking Soda
Herbs and spices
Tomato sauce, tomato paste
Relish
Margarine
Ready-to-eat frosting
Hunt's prima salsa spaghetti sauce
Buitoni mushroom spaghetti sauce
Old el Paso taco sauce
La Victoria green taco sauce
Jello pudding and pie filling (lemon, vanilla, butterscotch)
Royal pudding and pie filling (lemon, vanilla, butterscotch and coffee flavors)
Royal instant pudding and pie filling (lemon, vanilla, butterscotch and coffee flavors)
*If instructions call for 2 cups milk, substitute ¾ cup rich whip mixed with ¾ cup water.
<u>Only royal instant</u> pudding is allowed.

ALLOWED BEVERAGES

Tang (all flavors)
HiC fruit punches and fruit drinks
V-8 cocktail vegetable juice
Kook-Aid (all flavors)
Carbonated beverages (ex. Pepsi, Coke, Dr. Pepper, Gingerale, Sprite, etc.)

No beverages containing Aspartame are allowed.
